# TLR2 Activation by *Porphyromonas gingivalis* Requires Both PPAD Activity and Fimbriae

**DOI:** 10.3389/fimmu.2022.823685

**Published:** 2022-04-01

**Authors:** Aleksandra Wielento, Grzegorz P. Bereta, Katarzyna B. Łagosz-Ćwik, Sigrun Eick, Richard J. Lamont, Aleksander M. Grabiec, Jan Potempa

**Affiliations:** ^1^ Department of Microbiology, Faculty of Biochemistry, Biophysics and Biotechnology, Jagiellonian University, Krakow, Poland; ^2^ Department of Periodontology, Laboratory of Oral Microbiology, School of Dental Medicine, University of Bern, Bern, Switzerland; ^3^ Department of Oral Immunology and Infectious Diseases, School of Dentistry, University of Louisville, Louisville, KY, United States

**Keywords:** *Porphyromonas gingivalis*, citrullination, fimbriae, TLR2, reporter cell lines, gingival fibroblasts, peptidylarginine deiminase, inflammation

## Abstract

*Porphyromonas gingivalis*, a keystone oral pathogen implicated in development and progression of periodontitis, may also contribute to the pathogenicity of diseases such as arthritis, atherosclerosis, and Alzheimer’s. *P. gingivalis* is a master manipulator of host immune responses due to production of a large variety of virulence factors. Among these, *P. gingivalis* peptidilarginine deiminase (PPAD), an enzyme unique to *P. gingivalis*, converts C-terminal Arg residues in bacterium- and host-derived proteins and peptides into citrulline. PPAD contributes to stimulation of proinflammatory responses in host cells and is essential for activation of the prostaglandin E2 (PGE2) synthesis pathway in gingival fibroblasts. Since *P. gingivalis* is recognized mainly by Toll-like receptor-2 (TLR2), we investigated the effects of PPAD activity on TLR2-dependent host cell responses to *P. gingivalis*, as well as to outer membrane vesicles (OMVs) and fimbriae produced by this organism. Using reporter cell lines, we found that PPAD activity was required for TLR2 activation by *P. gingivalis* cells and OMVs. We also found that fimbriae, an established TLR2 ligand, from wild-type ATCC 33277 (but not from its isogenic PPAD mutant) enhanced the proinflammatory responses of host cells. Furthermore, only fimbriae from wild-type ATCC 33277, but not from the PPAD-deficient strains, induced cytokine production and stimulated expression of genes within the PGE2 synthesis pathway in human gingival fibroblasts *via* activation of the NF-ĸB and MAP kinase-dependent signaling pathways. Analysis of ten clinical isolates revealed that type I FimA is preferable for TLR2 signaling enhancement. In conclusion, the data strongly suggest that both PPAD activity and fimbriae are important for TLR2-dependent cell responses to *P. gingivalis* infection.

## Introduction

*Porphyromonas gingivalis* is a Gram-negative, anaerobic keystone oral pathogen implicated not only in the development and progression of periodontitis, but also in systemic diseases such as rheumatoid arthritis, atherosclerosis, and Alzheimer’s (1–3). *P. gingivalis* produces a large variety of virulence factors, among which lipopolysaccharide (LPS), gingipains, outer membrane vesicles (OMVs), fimbriae, capsules, and peptidylarginine deiminase (PPAD) play prominent roles (4). These virulence factors not only facilitate evasion of the host immune response by *P. gingivalis*, but also promote and sustain chronic inflammatory activation of host cells, which leads to breakdown of gingival tissue and release of nutrients that support the growth of *P. gingivalis* and other inflammophilic oral pathobionts (5).

PPAD is a unique virulence factor possessed by *P. gingivalis* and closely related periodontopathogenic *Porphyromonas* sp (6)., both of which belong to the *Porphyromonadaceae* family within the *Bacteroidales* order of *Bacteria*. The enzyme converts a C-terminal Arg residue in bacterium- and host-derived proteins and peptides into citrulline (7, 8). Our previous results demonstrate that PPAD activity is essential for activation of the prostaglandin E2 (PGE2) synthesis pathway in gingival fibroblasts (9), and for induction of proinflammatory genes in gingival epithelial cells (10). Nevertheless, no specific citrullinated *P.* gingivalis protein(s) that induce the host proinflammatory response were identified in proteome-wide studies of the *P.* gingivalis citrullinome (11, 12).

*P. gingivalis* is recognized by different pattern recognition receptors expressed by host cells; however, Toll-like receptor-2 (TLR2) plays a predominant role in this process. For example, production of proinflammatory cytokines such as TNF-α and IL-1β in response to live *P. gingivalis* is mostly dependent on TLR2 (13). In another study, TLR2-deficient mice were found to resist alveolar bone resorption following oral challenge with *P. gingivalis* (14). Furthermore, gingival tissue samples from periodontitis patients show higher expression of TLR2 than those from healthy individuals (15–17). Importantly, TLR2 is responsible for recognition of *P. gingivalis* fimbriae, which play a key role in bacterial adhesion to host cells, as well as in induction of proinflammatory signaling pathways (18–20).

In this study, we investigated how PPAD activity affects TLR2-dependent host cell responses to *P. gingivalis* infection. Using reporter cell lines and primary human gingival fibroblasts (PHGFs), we comprehensively compared the ability of various *P. gingivalis* strains and their isogenic mutants to activate TLR2. The results showed unambiguously that both PPAD activity and expression of major fimbriae are indispensable for TLR2 activation. Consistent with this, we also demonstrated that fimbriae isolated from the PPAD-deficient *P. gingivalis* mutant strain failed to activate TLR2, indicating that citrullination of fimbriae components or other proteins involved in fimbriae assembly is required for host cell activation through TLR2. Overall, our results highlight the importance of protein citrullination in the context of TLR2-dependent host responses and *P. gingivalis* virulence.

## Materials and Methods

### Cell Culture

Gingival biopsies were collected from healthy subjects presented for orthodontic treatment at the Department of Periodontology and Oral Medicine, Medical College, Institute of Dentistry, Jagiellonian University in Krakow, Poland. The study was approved by the Bioethical Committee of the Jagiellonian University in Krakow, Poland (permit number 122.6120.337.2016). Written informed consent was obtained from all donors in accordance with the Declaration of Helsinki. PHGFs were isolated from gingival tissue as described previously (21). PHGFs from different donors between passage 4 and 9 were used in experiments. PHGFs and U251 MG cells were cultured in DMEM (Gibco) supplemented with 10% FBS (Gibco), penicillin/streptomycin (50 U/ml) and gentamicin (50 U/ml), while HEK Blue hTLR2 cells (purchased from In vivogen) in DMEM with 10% FBS, penicillin/streptomycin (100 U/ml), Normocin™ (100 μg/ml) and HEK-Blue™ Selection In vivogen in 37°C, 5% CO_2_. Cells were seeded prior to experiments in medium without antibiotics supplemented with 2% FBS (PHGFs) or 10% FBS (U251 MG and HEK Blue hTLR2).

### Bacterial Culture, Cell Infection, and Treatment With TLR2 Ligands, Fimbriae, LPS, and OMVs

*P. gingivalis* including clinical strains, were grown anaerobically for 7 days on blood agar plates: brain-heart infusion (BHI, Becton-Dickinson) with addition of yeast extract containing 0.5 mg/mL L-cysteine, 10 µg/mL hemin, 0.5 µg/mL vitamin K and an appropriate antibiotic in the case of surface protein mutants (tetracycline for PPAD^C351A^, PPAD-T1 and PPAD-T2, erythromycin for delPPAD, delFimA and both tetracycline and erythromycin for double mutants: delFimA PPAD-T1, delFimA PPAD-T2 and delFimA PPAD^C351A^). Cells were infected with bacteria at early stationery growth phase (cultured in BHI with addition of above supplements without blood). Bacterial suspensions at OD_600_=1 (corresponding to 10^9^ colony-forming units/ml) prepared in PBS were used for infection. The MOI (multiplicity of infection) and time of infection used in each experiment are indicated in figure legends. Cells were also treated with the TLR2 ligand Pam3CSK4 (1 μg/ml), *P. gingivalis-* derived fimbriae (10 μg/ml), OMVs (2 μg/ml) or standard/ultrapure LPS (1 μg/ml; Invivogen. The duration of stimulation was analogous to the time of infection.

### *P. gingivalis* Mutant Strain Preparation

The construction of *P. gingivalis* PPAD^C351A^ and delPPAD strains was described in (9) and delFimA in (22). Strains expressing T1/T2 forms of PPAD and PPAD/FimA double mutants were prepared as follows. The coding sequence of PPAD (T1 form) together with flanking sequences was amplified from genomic DNA of *P. gingivalis* ATCC 33277 (primers FragA_FOR and REV-**Table 1**) the tetracycline resistance cassette with sequence downstream of *ppad* was amplified from genomic DNA of PPAD^C351A^ strain (primers FragB_FOR and REV- **Table 1**), pUC19 vector was linearized with PCR (primers pUC19_FOR and REV- **Table 1**). All fragments were assembled together with the Gibson Assembly method (New England Biolabs) to produce plasmid pUC_PPAD_T1. Point mutations G231N, E232T, and N235D (T2 form) were then introduced to the pUC_PPAD_T1 sequence in PCR reaction (primers MUT_FOR and REV-**Table 1**) and the plasmid was closed again with the Gibson Assembly reagent to produce plasmid pUC_PPAD_T2. Both plasmids were electroporated into ATCC 33277 wild-type or delFimA strains, 1 μg of plasmid DNA at 2.5 kV for 4 ms. Electrocompetent *P. gingivalis* was prepared using the method of Bélanger et al. (23). Following electroporation, bacteria were cultured on blood agar plates with tetracycline (1 µg/ml), and introduction of mutations was confirmed by sequencing.

**Table 1:** Sequences of primers used for PPAD T1/T2 mutants and a double PPAD-delFimA mutant construction.

Primer	Sequence
pUC19_FOR	GAGCTCGGTACCCGGGGATC
pUC19_REV	GAATTCACTGGCCGTCGTTTTACAACG
FragA_FOR	ACGGCCAGTGAATTCTTTACGGGCGGTTATCGGG
FragA_REV	GAGATAATTCGTTGTATTAAGAATATCAGTGGAGAAAATAATAC
FragB_FOR	ACAACGAATTATCTCCTTAACG
FragB_REV	CCGGGTACCGAGCTCCTCCGTATAGAGCAGGATC
MUT_FOR	AACAATACTTATATCGACCATGTGGACTGTTGGGGCAAGTATTTGGC
MUT_REV	CACATGGTCGATATAAGTATTGTTCGGATCTTGTACCACATCATGATGTGTGATGC

### Outer Membrane Vesicles (OMVs) Isolation

Bacteria were grown in 80 ml culture to the late exponential phase. After centrifugation at 6, 000 x g, 40 min, 4°C, growth media were collected and filtered through 0.22-μm-pore-size filters. Vesicles were collected by ultracentrifugation at 100,000 x g, 1 h, 4°C. Pellets were suspended in approx. 0.5 ml PBS and gently sonicated to make a uniform suspension. Aliquots of OMVs were stored in -80°C.

### Fimbriae Isolation

Fimbriae were isolated as described in (24) with some modification. Briefly, bacteria were grown in 2 L culture to OD_600_= 1.2-1.4 and then harvested by centrifugation at 8, 000 x g, 20 min. The pellet was suspended in approx. 1/10 the original volume of the culture in 20 mM Tris-HCl, 0.15 M NaCl, 10 mM MgCl_2_, pH 7.4, by repeated pipetting. After agitation of the suspension for 30 min on a magnetic stirrer, bacterial cells were removed by centrifugation (8,000 x g, 20 min) and the supernatant (bacterial wash) collected. To avoid cell lysis, all precipitation steps were performed at room temperature. Proteins in the bacterial wash were precipitated with 40% saturated ammonium sulfate, precipitate was collected by centrifugation at 20,000 x g, 20 min, 4°C and then suspended in small volume of 20 mM Tris-HCl, pH 8.0. After overnight dialyses against 2 x 1.5 L of 20 mM Tris-HCl, pH 8.0, the dialysate was clarified by centrifugation (20,000 x g, 20 min, 4°C) and applied to a DEAE-Sepharose ion exchange column equilibrated with the above buffer. The column was washed with 20 mM Tris-HCl, pH 8.0 (until OD_280_ dropped below 0.05) and protein elution was performed with 0 – 0.5 M NaCl gradient, where fimbriae emerged in the middle of the gradient. Fractions containing protein were pooled and precipitated with 40% saturated ammonium sulfate as previously. Protein was suspended in smallest possible volume of PBS and dialyzed against 3x 1 L PBS. SDS-PAGE was run for purity verification. Fimbriae were stored at -80°C.

### RNA Isolation and Quantitative (q)PCR

Total RNA was extracted using ExtractMe Total RNA isolation kit (Blirt) and quantified with a Nanodrop spectrophotometer (Thermo Scientific). RNA was reverse-transcribed using a High-Capacity cDNA Reverse Transcription Kit (Applied Biosystems). PowerUp SybrGreen PCR mix (Applied Biosystems) was used to perform quantitative PCR. mRNA expression level relative to β-actin was analyzed using CFX Manager (Bio-Rad). The following sequences of primers were used: β-actin F (CCACACTGTGCCCATCTACG), β-actin R (AGGATCTTCATGAGGTAGTCAGTCAG), mPGES-1 F (CACGCTGCTGGTCATCAAGAT), mPGES-1 R (CCGTGTCTCAGGGCATCCT), COX-2 F (AGCCCTTCCTCCTGTGCCT), COX-2 R(AATCAGGAAGCTGCTTTTTACCT).

### Reporter Assays

U251 MG cells were seeded on 24-well plates in DMEM containing 10% FBS. The next day cells were transiently transfected with: (i) a vector (pGL2-NFκB, 247.5 ng per well) coding the *Firefly* luciferase gene under control of 5 tandem repeats of the NF-ĸB response element, (ii) a reference pEF vector (5 ng per well) coding β-galactosidase under control of EF-1α elongation factor, and (iii) a vector (247.5 ng per well) encoding the human flag-tagged TLR2 receptor or TLR2-TLR1/TLR2-TLR6 heterodimer (pDUO-hTLR1-TLR2/pDUO-hTLR1-TLR2, Invivogen or an empty vector pcDNA3.1, in total 500 ng per well. The vector encoding the human flag-tagged TLR2 receptor was a gift from Ruslan Medzhitov (Yale University, USA, Addgene plasmid #13082; http://n2t.net/addgene:13082; RRID : Addgene_13082). Transfections were performed using PEI MAX 40000 (Polysciences) at a reagent to DNA ratio of 3:1. At 24 h posttransfection, culture medium was replaced with DMEM containing 0.5% FBS and cells were infected with *P. gingivalis* or stimulated with TLR2 ligands. After 4 h, cells were lysed in PLB buffer (Promega). *Firefly* luciferase and β-galactosidase activities were measured using Dual-Glo and Beta-Glo assay (both from Promega), respectively. To correct for transfection efficiency, luciferase activity was calculated as a ratio of *Firefly* luciferase activity to β-galactosidase activity. Results from cells transfected with the vector encoding TLR2 were also normalized to results from cells transfected with the empty vector and stimulated/infected with the same factor.

HEK-Blue hTLR2 cells with the stably integrated NF-κB-inducible SEAP reporter gene expressing alkaline phosphatase after TLR2 activation were seeded in 96-well plates in DMEM supplemented with 10% FBS. The following day, culture medium was replaced with DMEM containing 0.5% FBS and cells were infected or stimulated with TLR2 ligands for 4 h. Supernatants were collected and SEAP activity was measured using QUANTI-Blue™ Solution (Invivogen) according to the manufacturer’s instructions.

### Western-Blot

Cells were lysed in Laemmli’s buffer containing 2% SDS, 10% glycerol and 125 mM Tris-HCl, pH 6.8. A Bradford assay was used to determine protein concentration in cell lysates. Equal amounts of protein were analyzed by Western-blot as described previously (25). Specific bands corresponding to a protein of interest were detected by primary antibodies against p65 (#8242), p-p65 (#3033), p-p38 (#9211) and β-actin (#4967) (all from Cell Signaling Technology) or antisera against FimA (26), and horseradish peroxidase (HRP)–conjugated anti-rabbit Ig secondary antibodies (Dako). Blots were developed using a ClarityWestern ECL Substrate (Bio-Rad) and visualized using a ChemiDocMP Imaging System and the ImageLab software (Bio-Rad).

### PPAD Activity Assay

PPAD activity was assessed in suspensions of washed bacterial cells adjusted to OD_600_= 1 as described previously (27). Briefly, cell suspensions were incubated for 1 h with substrate (10 mM Nα-acetylarginine) in 100 mM Tris-HCl, pH 7.5 supplemented with 5 mM 1,4-dithiothreitol to initiate the reaction. The reaction was stopped with 5 M perchloric acid and absorbance at 535 nm was measured using a microplate reader (Molecular Devices).

### ELISA

PHGFs were treated with purified fimbriae (10 µg/ml) for 24 h. Culture media were then collected, and the levels of IL-6 and IL-8 were determined using commercially available ELISA MAX Standard sets (BioLegend) according to the manufacturer’s instructions.

### *fimA* and *fimB* Gene Sequencing

Genomic DNA was isolated from *P. gingivalis* clinical strains using Genomic Mini kit (A&A Biotechnology). The *fimA* and *fimB* genes were amplified using Phusion™ High-Fidelity DNA Polymerase (Thermo Fischer) with primers: fimA uni-F (AAGTTTTTCTTGTTGGGACTTGC), fimA uni-R (AACCCCGCTCCCTGTATTCCGA) (28) and fimB F (ATCGTATCGGTGCTGATCTTACTCG), fimB R (TCTGCATATTGTTGCACTACGTCCC). The amplification cycles were as follows: 98°C 30s initial denaturation, then 35 cycles of denaturation, annealing and extension 98°C 10s, 68°C 30s and 72°C 30s, respectively, and final extension 72°C, 10 min, 4°C hold. PCR products were run in 1% agarose gels containing ethidium bromide and bands corresponding to the *fimA* or *fimB* gene size were extracted from the gel using GeneJET Gel Extraction Kit (Thermo Fischer). The *fimA* or *fimB* gene was then cloned into the pJET plasmid (Thermo Fischer). Recombined plasmids were propagated in *E. coli* DH5α strain and isolated using GeneJET Plasmid Miniprep Kit (Thermo Fischer). All kits were used according to manufacturer’s instructions. Isolated plasmids were sequenced by Genomed, Poland and results were analyzed using Needle (EMBOS-EBI) and Chromas Lite (Technelysium Pty Ltd) programs.

### Statistical Analyses

Data are presented as mean ± SD unless otherwise indicated. All experiments on reporter cell lines were conducted at least three times and the exact numbers of repeats of each experiment are included in the figure legends. For experiments performed on PHGFs, the values of “n” refer to the number of cell lines isolated from different donors that were used in each experiment. One-way analysis of variance (ANOVA) followed by the Tukey's multiple comparison test was used for analyzing the data unless otherwise indicated. A probability (*p*) value of <0.05 was considered statistically significant. Statistical analysis was performed with GraphPad Prism 8.02 (GraphPad Software, Inc.).

## Results

### *P. gingivalis* Stimulates Proinflammatory Responses in the U251 MG Cell Line in a TLR2-Dependent Manner

**Figure 1:**
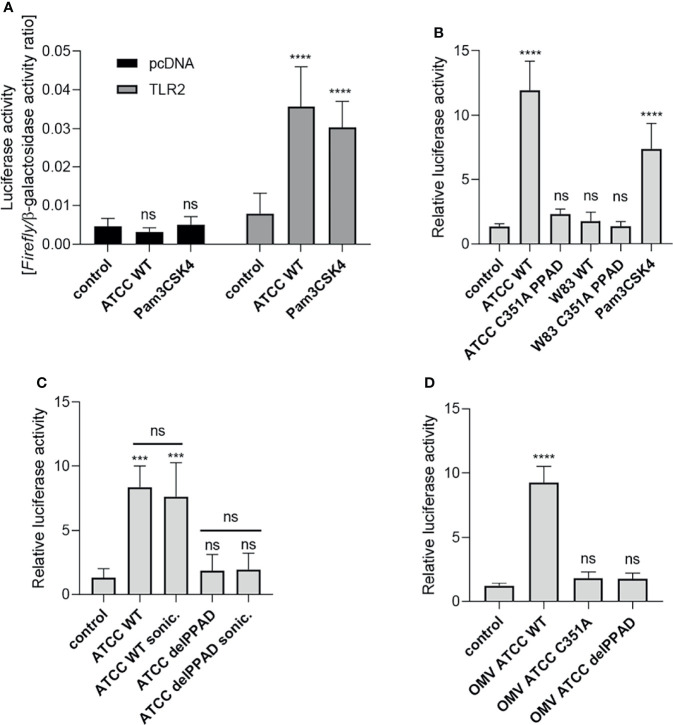
Activation of TLR2 is dependent on PPAD expression and activity. U251 MG cells overexpressing the TLR2 receptor were infected (MOI=100) or treated for 4 h with **(A)** ATCC 33277 strain or Pam3CSK4 (1 μg/ml), n=5-7; **(B)** various *P. gingivalis* strains and ATCC-derived isogenic mutants of catalytically inactive PPAD (C351A PPAD), n=3-5; **(C)** ATCC 33277 wild-type and PPAD mutant strains and sonicates (cells were lysed by sonication (sonic.)), n=4; or **(D)** outer membrane vesicles (OMVs) isolated from ATCC 33277 strain and its isogenic PPAD mutants (2 µg/ml), n=4. Results in **(A)** are presented as a ratio of *Firefly* luciferase activity to β-galactosidase activity. Results in **(B–D)** are presented as a ratio of *Firefly* luciferase activity to β-galactosidase activity. Data are normalized to those from cells stimulated/infected with the same factor and transfected with an empty vector. Mean + SD; ****p < 0.0001; ***p < 0.001; ns, no statistical significance; 1-way ANOVA both followed by Tukey’s multiple comparisons test. ATCC WT- ATCC 33277.

Our previous work showed that PPAD is essential for *P. gingivalis*-induced inflammatory responses by PHGFs, although the underlying mechanism(s) remain unknown (9). Since TLR2 is the key receptor engaged by *P. gingivalis* (14, 29), we evaluated how PPAD expression and activity affect TLR2-dependent responses. First, we established a reporter system (U251 MG-hTLR2) using the U251 MG cell line co-transfected with a NF-κB-dependent reporter (*Firefly* luciferase) and TLR2 coding vectors. Of note, U251 MG cells express undetectable level of TLR2 (25) and have very low constitutive expression of TLR4 (Human Protein Atlas). Cells transfected with the empty vector (pcDNA) had low constitutive luciferase activity, which was unaffected by infection with *P. gingivalis* or stimulation with the TLR2 ligand Pam3CSK4. By contrast, TLR2-expressing cells showed a significantly greater increase in luciferase activity upon exposure to *P. gingivalis* or Pam3CSK4 than untreated cells, in which luciferase activity was comparable with that in cells transfected with the empty vector (**Figure 1A**). Therefore, in all subsequent experiments performed on U251 MG-hTLR2 cells, luciferase activity was normalized to the constitutive background bioluminescence in cells without TLR2 overexpression.

### Activation of TLR2 Is Dependent on PPAD Expression and Activity

Using the established assay, we investigated the requirement for PPAD activity during TLR2-dependent stimulation of the NF-κB pathway by *P. gingivalis* strains W83 and ATCC 33277. U251 MG-hTLR2 cells were infected with wild-type (WT)-*P. gingivalis* strains and mutants expressing catalytically inactive PPAD (PPAD^C351A^). Pam3CSK4 was used as a positive control. Interestingly, only WT-*P. gingivalis* ATCC 333277 induced luciferase expression (**Figure 1B**). This was reduced to background levels in cells infected with the PPAD^C351A^-ATCC 33277 strain. The importance of PPAD for TLR2 activation in response to the ATCC 33277 strain was confirmed independently using HEK Blue hTLR2 cells (**Supplementary Figure 1A**). Since TLR2 signals not only as a homodimer, but also forms heterodimers with TLR1 and TLR6 (30), we tested how expression of these co-receptors in U251 MG reporter cells affects *P. gingivalis*-induced TLR2 signaling. We found that the ATCC 33277 strain activated the TLR2 homodimer as well as the TLR2-TLR1 and TLR2-TLR6 heterodimers. Although engagement of the TLR2 homodimer caused the most robust induction of luciferase expression, in all three cases reporter cell activation was PPAD-dependent (**Supplementary Figure 1B**). The results suggest that citrullination of a protein, or proteins, unique to ATCC 33277 is responsible for induction of TLR2 signaling.

To explore the subcellular location of this putative signaling ligand, we compared the responses of U251 MG-hTLR2 cells to intact *P. gingivalis*, bacterial cell lysates (**Figure 1C**), and isolated OMVs produced by the parental strain and the PPAD-deficient mutants delPPAD and PPAD^C351A^ (**Figure 1D**). In all cases, luciferase induction was dependent on PPAD activity, regardless of the fraction tested. The lack of difference in TLR2 activation elicited by intact bacteria and bacterial sonicates (**Figure 1C**) suggests absence of an intracellular reservoir of TLR2 ligands that are dependent on citrullination. This is consistent with robust activation of the reporter gene by WT-OMVs (**Figure 1D**), which are considered to be surrogates of the outer membrane and have been shown to contain PPAD (31, 32). These results were confirmed using HEK Blue hTLR2 cells, which responded with an 8-fold increase in phosphatase activity upon treatment with WT ATCC 33277-derived OMVs, but not with OMVs produced by PPAD-deficient mutants (**Supplementary Figure 1C**). These results indicate that TLR2 activation by *P. gingivalis* ATCC 33277 depends on the activity of PPAD, which appears to citrullinate some cell surface proteins that function as TLR2 ligands. The lack of signaling by the W83 strain in U251 MG-hTLR2 cells is likely due to the absence of the protein(s) susceptible to modification by PPAD.

### Fimbriae From Wild-Type but Not From PPAD-Deficient *P. gingivalis* Activate TLR2

**Figure 2:**
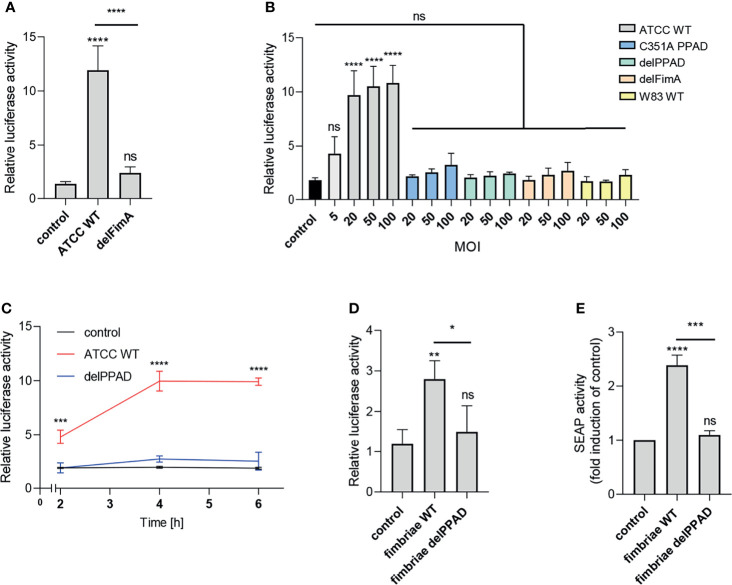
Fimbriae purified from the wild-type ATCC 33277 strain activate the TLR2 receptor. U251 MG cells overexpressing TLR2 were: **(A)** infected for 4 h with WT ATCC 33277 and its isogenic major fimbriae (delFimA) mutant strain (MOI=100), n=4-5 **(B)** infected for 4 h with various ATCC 33277-derived PPAD and FimA mutants as well as the WT W83 strain at different MOI (all strains MOI=20-100 with an additional MOI=5 for WT ATCC 33277), n=3 or **(C)** infected for 2, 4 or 6 h with WT ATCC 33277 or the PPAD mutant strains (MOI=100), n=3; and **(D)** treated for 4 h with purified fimbriae (10 µg/ml) from WT ATCC 332771 (FimA WT) or the PPAD mutant (FimA delPPAD) strains; n=4. Results are presented as the mean ± SD ratio of *Firefly* luciferase activity to β-galactosidase activity and are normalized to cells stimulated/infected with the same factor and transfected with an empty vector. **(E)** HEK Blue cells overexpressing TLR2 were treated with purified fimbriae (10 µg/ml) isolated from the WT ATCC 33277 strain (FimA WT) or the PPAD mutant (FimA delPPAD), n=3. Results are presented as the mean ± SD fold induction compared to control (unstimulated) cells. ****p < 0.0001; ***p < 0.001; **p < 0.01; *p < 0.05; ns, no statistical significance. 1-way ANOVA followed by Tukey’s multiple comparisons test. In **(B)** each condition was compared to control uninfected cells and in **(C)** comparisons were performed for each timepoint separately and significant differences compared to WT ATCC 33277 are depicted in the graph.

The most apparent difference between the *P. gingivalis* ATCC 33277 and W83 strains is the presence of fimbriae on the surface of the former and the lack thereof on the latter (33). Therefore, we hypothesized that these bacterial cell surface appendages may be a potential source of a PPAD-dependent TLR2 ligand activation. To verify this possibility, we infected cells with the ATCC 33277-derived mutant of the major fimbriae subunit (delFimA). In line with our expectations, the delFimA mutant lacked the ability to activate TLR2, suggesting that FimA is recognized by TLR2 and activates the NF-κB pathway in U251 MG-hTLR2 cells (**Figure 2A**).

Activation of the reporter cells by the parental ATCC 33277 strain was dose- and time-dependent. A significant response was observed with MOI = 5, reaching maximum activation at MOI = 20 (**Figure 2A**), and was more pronounced in cells exposed to bacteria for 4 h and 6 h compared to infection for 2 h (**Figure 2C**). Conversely, cells infected with *P. gingivalis* W83, delPPAD and delFimA responded only with a minor, albeit MOI-dependent, increase of luciferase activity, which was statistically insignificant (**Figure 2B**).

To confirm that PPAD-modified fimbriae are responsible for signaling through TLR2, we treated U251 MG-hTLR2 cells with purified fimbriae produced by parental ATCC 33277 and the delPPAD strain. Of note, western blot analysis of purified fimbriae as well as OMVs from wild type and PPAD mutant strains yielded the same band pattern (**Supplementary Figures 2A, B**), suggesting that the lack of PPAD did not affect fimbriae assembly. Both reporter cell lines responded only to treatment with fimbriae from the parental strain, not from delPPAD *P. gingivalis* (**Figures 2D, E**). Since there are some reports in the literature that *P. gingivalis* LPS can signal through TLR2 (34), we determined how LPS alone or in combination with fimbriae affects luciferase activity in U251 MG-hTRL2 cells. Two types of *P. gingivalis* LPS preparations, which are distinguished by the presence or absence of lipoprotein contamination, are commonly used for such studies (35). We found that neither ultrapure nor standard preparations of *P. gingivalis* LPS increased luciferase activity significantly. Similarly, LPS had no significant effect on fimbriae-induced TLR2 activation, although treatment with fimbriae in combination with a standard LPS preparation was slightly more potent than fimbriae alone with respect to reporter gene activation (**Supplementary Figure 2B**). The very weak response to a standard LPS preparation can be explained by an absence of accessory proteins that enhance TLR2 signaling. Indeed, the expression of CD14 and CD36, which participate in ligand delivery to TLR2 (30) is low in U251 MG cells according to the Human Protein Atlas. Taken together, these results indicate that *P. gingivalis* LPS does not act synergistically with fimbriae in the reporter cell line, further confirming that PPAD-modified fimbriae are the main ligand activating the TLR2 signaling pathway.

### The Level of TLR2 Activation Is Independent of the Presence of PPAD Activity Variants

**Figure 3:**
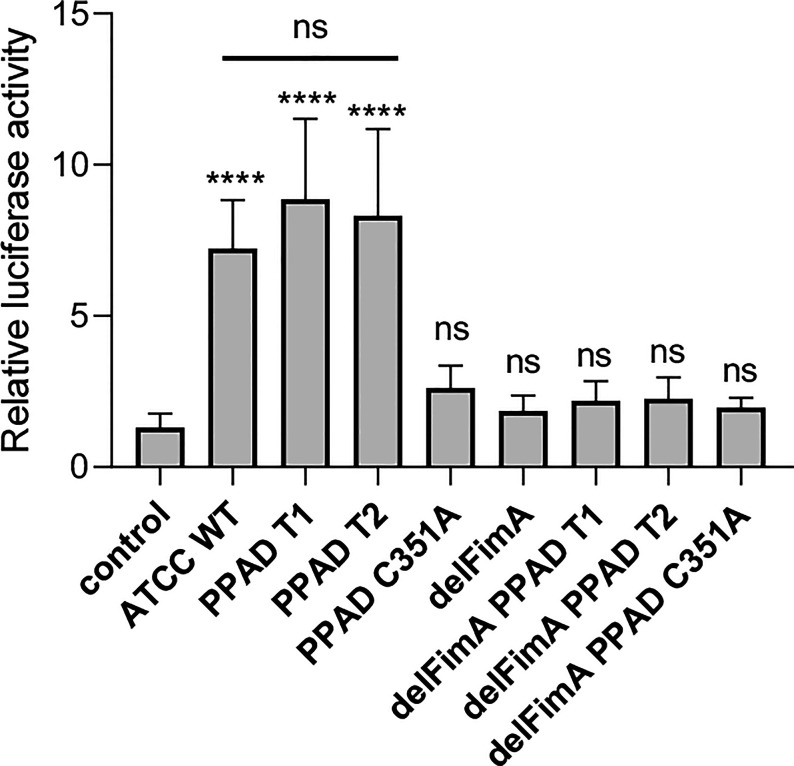
Both active PPAD and fimbriae are crucial for activation of TLR2. U251 MG cells overexpressing the TLR2 receptor were infected for 4 h (MOI=100) with various ATCC 33277-derived isogenic mutants expressing different forms of PPAD (the T1 form, the T2-hyperactive form, and the catalytically inactive C351A mutant form) or FimA; n=4. Results are presented as the mean ± SD ratio of *Firefly* luciferase activity to β-galactosidase activity and are normalized to cells stimulated/infected with the same factor and transfected with an empty vector. ****p < 0.0001; ns, no statistical significance; 1-way ANOVA followed by Tukey’s multiple comparisons test.

Two forms of PPAD, which differ in terms of their catalytic potency due to amino acid substitution in the substrate binding cleft, are present in *P. gingivalis* strains (36). To determine whether PPAD variants affect fimbriae signaling *via* TLR2 differently, we compared the response of U251 MG-hTLR2 cells infected with the ATCC 33277 strain expressing normal (PPAD-T1) with that of the super-active (PPAD-T2) form of the enzyme. We found no difference in luciferase induction by the PPAD-T1- and PPAD-T2-expressing strains (**Figure 3**). As expected, PPAD^C351A^, delFimA, delFimA/PPAD-T1, and delFimA/PPAD^C351A^ elicited no activation of the reporter gene. Remarkably, infection with delFimA/PPAD-T2 caused no increase in luciferase activity in infected cells (**Figure 3**), despite possessing 2-fold higher PPAD activity than delFimA/PPAD-T1 (36). This finding indicates that both forms of PPAD are equally efficient in inducing fimbriae modifications essential for TLR2 signaling.

### Clinical *P. gingivalis* Isolates Activate TLR2-Dependent Signaling Poorly

**Figure 4:**
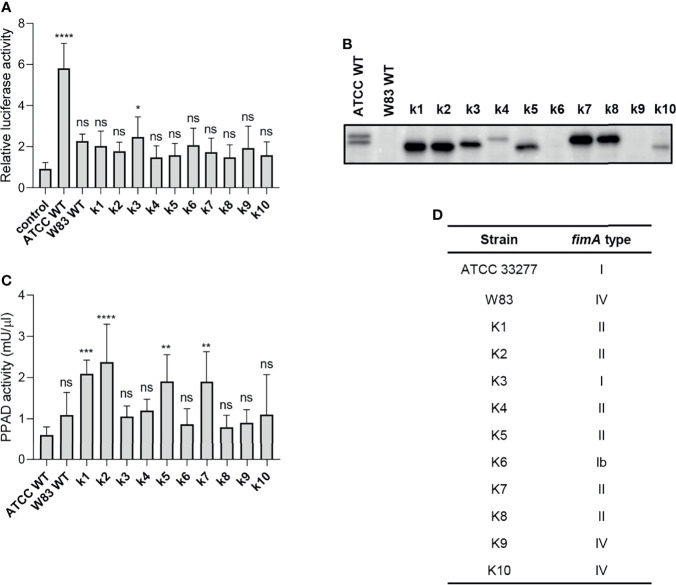
The ability of clinical strains to activate TLR2 is much weaker than that of ATCC 33277. **(A)** Cells were infected for 4 h (MOI=100) with various clinical strains (k1-k10), ATCC 33277 (ATCC WT), or W83, n=4. Results are presented as the mean ± SD ratio of *Firefly* luciferase activity to β-galactosidase activity and are normalized to cells stimulated/infected with the same factor and transfected with an empty vector. **(B)** Western blot analysis of laboratory and clinical strain cultures (adjusted to OD_600_) to detect FimA. **(C)** PPAD activity in whole laboratory and clinical strain cultures (adjusted to OD_600_), n=6. Results were compared with those from the ATCC 332777 strain. *p < 0.05; **p < 0.01; ***p < 0.001; ****p < 0.0001; ns, no statistical significance. 1-way ANOVA followed by Tukey’s multiple comparisons test. **(D)** The FimA type determined by sequencing of the *fimA* gene in clinical strains.

To explore *P. gingivalis* strain-dependent TLR2 signaling, we tested a series of clinical *P. gingivalis* isolates for their ability to activate U251 MG-hTLR2 cells. Interestingly, out of ten tested strains, only one (K3) increased luciferase activity in infected cells significantly, albeit to a much lesser extent than WT ATCC 33277 (**Figure 4A**). To determine the cause of these differences, we analyzed fimbriae expression (**Figure 4B**) and PPAD activity (**Figure 4C**) in all tested clinical strains. In both cases, we noted large variations between the examined strains, as well as between the clinical and laboratory strains, which showed no apparent relationship with their ability to activate TLR2.

Since individual *P. gingivalis* strains may carry one of six different alleles of the *fimA* gene (37), we hypothesized that expression of type I FimA, which is present in the ATCC 33277 strain, could be linked specifically to TLR2 signaling. Indeed, sequencing of the *fimA* gene from clinical strains revealed a high prevalence of type II and IV *fimA*, although the K3 and K6 strains expressed type I and Ib *fimA*, respectively (**Figure 4D**). Selective stimulation of TLR2 signaling by the ATCC 33277 and K3 strains bearing type I FimA suggests that this type of FimA could be the preferred ligand for TLR2 activation, at least in the reporter cell lines used in this study. Interestingly, K3 (the only clinical isolate able to activate TLR2) displayed intermediate expression of fimbriae (**Figure 4B**) and PPAD activity (**Figure 4C**), suggesting that a specific type of fimbriae must be expressed in the presence of PPAD activity to fully activate TLR2. It is noteworthy that the cell-stimulatory potential also depends on the FimB-subunit affecting the length of the fimbriae. The ATCC 33277 strain possesses a premature STOP codon in the *fimB* gene, which makes its fimbriae aberrantly long and highly stimulating to cells (38). For this reason, we sequenced the *fimB* gene in clinical isolates and found that all tested strains encode functional FimB (data not shown). This may explain, at least partly, the weak immunostimulatory potential of the clinical isolates.

### A Lack of PPAD or FimA Reduces Inflammatory Responses by PHGFs

**Figure 5:**
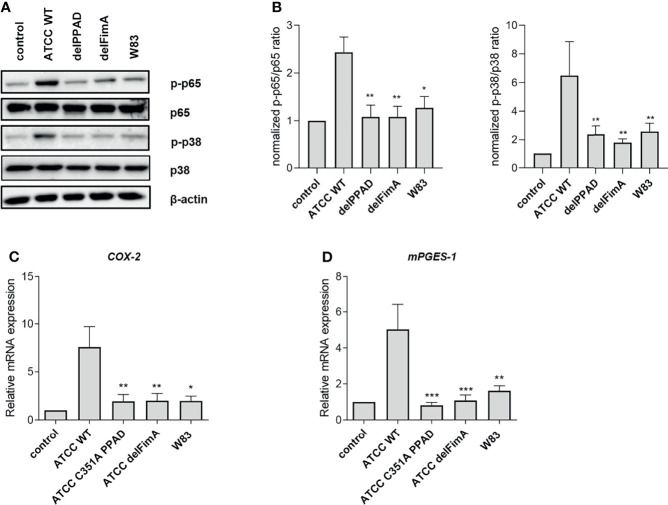
Activation of the NF-ĸB and MAPK kinase pathways, and expression of PGE2 synthesis pathway-related genes, are induced by citrullinated, fimbriated *P. gingivalis* in PHGFs. PHGFs were infected for 1 h with the WT ATCC 33277 strain (ATCC WT) and its isogenic PPAD (delPPAD) and FimA mutants (delFimA), or the WT W83 strain at an MOI=20. **(A, B)** Western blot analysis was performed to detect total and phosphorylated p65 and p38. β-actin was used as a control. **(A)** Representative blots and **(B)** densitometry analysis of n=4 independent experiments is shown as the mean ± SEM. Relative mRNA expression of PGE2 synthesis-related genes **(C)** COX-2 and **(D)** mPGES-1 in PHGFs (n=10) infected with various *P. gingivalis* mutants. Cells were infected for 24 h at an MOI=100. Data represent the mean ± SEM **(B)** normalized phosphorylated to total protein ratio or **(C, D)** relative mRNA expression; ***p < 0.001; **p < 0.01; *p < 0.05; 1-way ANOVA followed by Tukey’s multiple comparisons test; data from the ATCC 33277 strain were compared to all other strains; “n” represents the number of independent experiments performed on PHGFs cell lines derived from different healthy donors.

To verify if the results obtained using reporter cell lines can be reproduced in primary cells, we performed experiments using PHGFs, which are an important source of inflammatory mediators in gingival tissues (39) and respond to *P. gingivalis* infection predominantly through TLR2 (25). Since engagement of TLRs initiates the NF-κB and mitogen-activated protein kinase (MAPK) signaling pathways (40), we investigated the activation status of these pathways upon infection with various *P. gingivalis* strains and mutants. As expected, WT ATCC 33277 induced robust phosphorylation of p38 MAP kinase and the p65 subunit of NF-κB, whereas the delPPAD and delFimA mutants, as well as the WT W83 strain, had negligible effects on these pathways (**Figures 5A, B**). Consistent with this, expression of prostaglandin E2 (PGE_2_) synthesis pathway genes *COX-2* and *mPGES-1* increased significantly in the presence of the WT-ATCC 33277 strain compared with the WT-W83 strain and the delPPAD and delFimA mutant strains (**Figures 5C, D**).

**Figure 6:**
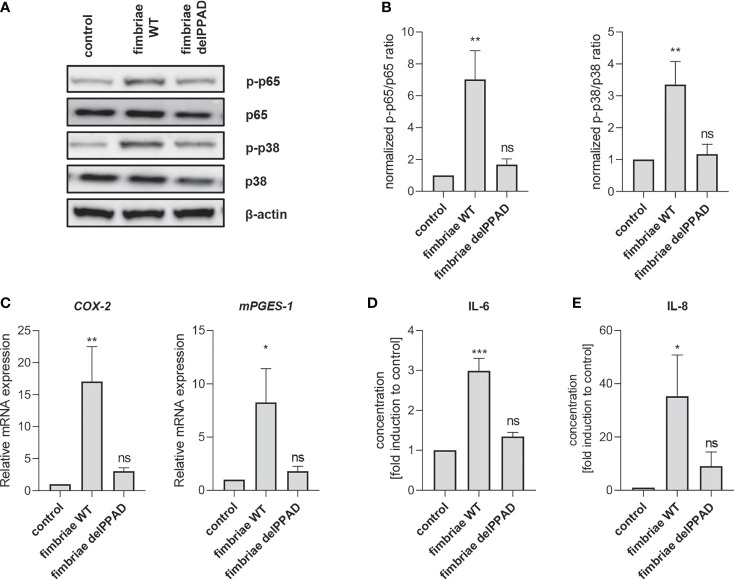
Fimbriae purified from the WT-ATCC 33277 strain stimulate PHGFs to produce cytokines and activate the NF-ĸB and MAPK kinase signaling pathways. **(A, B)** Western blot analysis of phosphorylated and total p65 and p38 in PHGFs (n=7) treated for 1 h with purified fimbriae (10 µg/ml) isolated from WT-ATCC 33277 (FimA WT) or its isogenic PPAD mutant (FimA delPPAD). β-actin was used as a control. **(A)** Representative blots and **(B)** results of densitometry analysis are shown. **(C)** Expression of mRNA encoding PGE2 synthesis-related genes COX-2 and mPGES-1 in PHGFs (n=7) treated for 4 h with purified fimbriae (10 µg/ml) isolated from WT-ATCC 33277 strain (FimA WT) or its isogenic PPAD mutant (FimA delPPAD). **(D, E)** Secretion of IL-6 **(D)** and IL-8 **(E)** by PHGFs (n=5) treated for 24 h with purified fimbriae (10 µg/ml) from WT and delPPAD ATCC 33277 strains. Data represent the mean ± SEM normalized phosphorylated to total protein ratio **(B)**, relative mRNA expression **(C)** or fold change of cytokine concentration **(D, E)**; ***p < 0.001; **p < 0.01; *p < 0.05; ns, no statistical significance; 1-way ANOVA followed by Tukey’s multiple comparisons test; “n” represents the number of independent experiments performed on PHGFs cell lines derived from different healthy donors.

To confirm the essential role of PPAD activity in fimbriae-mediated PHGF inflammatory activation, we treated the cells with fimbriae purified from WT-ATCC 33277 and the delPPAD mutant strain, and then measured the activation status of intracellular signaling pathways and changes in inflammatory gene expression. Consistent with the pattern of PHGF responses to WT and mutant *P. gingivalis* strains, fimbriae from WT-*P. gingivalis* were more potent in inducing phosphorylation of p38 MAP kinase and the p65 NF-κB subunit than those from the PPAD-deficient mutant strain (**Figures 6A, B**). Additionally, qPCR analysis of *COX2* and *mPGES1* expression revealed that fimbriae from WT-ATCC 33277 were significantly better at enhancing the PGE2 synthesis pathway than fimbriae from the PPAD-null strain (**Figure 6C**). Finally, fimbriae from WT-*P. gingivalis* induced PHGFs to secrete much greater amounts of IL-6 and IL-8, whereas fimbriae from the PPAD-null mutant upregulated production of these cytokines to a much lesser extent (**Figures 6D, E**).

Collectively, our results show that both PPAD activity and fimbriae are required for TLR2 activation and that their presence is indispensable for enhancement of host proinflammatory responses by *P. gingivalis*. Although citrullination of fimbriae was not confirmed formally, the data clearly show a direct link between PPAD and fimbriae in the context of TLR2 activation.

## Discussion

*P. gingivalis* produces a wide array of virulence factors that manipulate host immune responses. The bacterium alters both the environment and host-induced signaling to support its fitness by promoting inflammophilic conditions and avoiding elimination by the host immune system (2). Herein, we identified a direct connection between two important *P. gingivalis* virulence factors, PPAD and fimbriae, and demonstrate that simultaneous presence of both factors is required for activation of TLR2-dependent host signaling. To date, post-translational modifications of fimbriae have not been analyzed comprehensively, and studies of citrullination focused predominantly on host proteins rather than bacterial factors (8, 41). We show that whole bacterial cells, OMVs, and fimbriae derived from PPAD-deficient strains of *P. gingivalis* lack TLR2-stimulatory potential, indicating that citrullination of bacterial proteins is necessary for TLR2 engagement by *P. gingivalis*.

We show for the first time that fimbriae derived from the PPAD-deficient mutant have significantly reduced potential to stimulate TLR2 signaling in reporter cell lines and to induce inflammatory activation of PHGFs. In this respect, they mimic the effects of infection with the FimA- and PPAD-deficient mutants. This raises the possibility that FimA or other fimbriae subunits can undergo PPAD-mediated citrullination, which is prerequisite for binding to and activation of TLR2. Unfortunately, data on fimbriae modification by PPAD are scarce; nevertheless, citrullination of FimA type II was found in OMVs produced by *P. gingivalis* clinical isolates associated with rheumatoid arthritis, an inflammatory joint disease in which anticitrullinated protein antibodies contribute significantly to pathology (11). By contrast, despite the presence of PPAD in OMVs (32), mass spectrometric analysis failed to detect citrullination of FimA in ATCC 33277-derived OMVs (11) and in the *P. gingivalis* 381 strain (42). This, however, does not exclude the possibility that FimA undergoes citrullination, since detection of citrullinated peptides is technically challenging, and strain-specific differences in citrullination efficiency are likely. Worthy of note is that we tried to complement fimbriae with active purified PPAD using our reporter cell system with no success (data not shown). The failed complementation is likely the result of modification occurring during assembly of the fimbriae. This is supported by colocalization of PPAD and Arg-gingipains on the *P. gingivalis* surface (32, 43, 44). Apparently once fimbriae subunits are assembled into the shaft, they are no longer susceptible to modification by PPAD.

In addition to FimA, the major component forming a shaft, *P. gingivalis* fimbriae contain accessory proteins FimC, FimD, and FimE, which may be a target for PPAD-catalyzed citrullination prior to interaction with TLR2. In this regard it is noteworthy that the FimA-deficient mutant also lacks the accessory fimbriae subunits (45). The possibility that PPAD-mediated modification of accessory fimbriae subunits may significantly contribute to TLR2 activation is supported by the findings of Hajishengallis et al who demonstrated that the ATCC 33277 isogenic mutant OZ5001C expressing DAP fimbriae (devoid of all three accessory proteins and expressing only FimA) is less virulent than the parental strain (46). DAP fimbriae bind preferentially to only one TLR2 molecular partner, TLR1, while native fimbriae are able to recruit both TLR1 and TLR6 (20). Additionally, DAP fimbriae, in contrast to native fimbriae, do not interact with the CXCL12 chemokine receptor CXCR4, which participates in receptor crosstalk with TLR2 and facilitates *P. gingivalis* escape from host recognition and killing (47). These observations highlight the multiple roles of major fimbriae in *P. gingivalis* interactions with host cells. Since our mass spectrometric analysis of fimbriae preparations revealed the presence of accessory subunits (data not shown), we cannot exclude the possibility that one or more accessory major fimbriae components are subject to modification by PPAD. Moreover, in addition to the major fimbriae, *P. gingivalis* also express minor (short) fimbriae (37). These structures participate in auto-aggregation and biofilm formation, and their assembly and structure are similar to the major fimbriae (48). Further, the main subunit, Mfa1, which is not expressed in the W83 strain (43), has been shown to activate fibroblasts in a partially TLR2-dependent manner (26, 49). This raises the possibility that not only major fimbriae components, but also minor fimbriae could undergo modification by PPAD which modulates their TLR2-stimulating potential. We will investigate this possibility in future studies.

It is also important to note that *P. gingivalis*-induced TLR2 signaling can converge on the MyD88 adaptor-dependent classical pathway, leading to NF-ĸB activation, or can be MyD88-independent, resulting in activation of the PI3K/AKT pathway (14, 19). Supposedly, citrullinated ligands are better inducers of the MyD88-dependent pathway, while unmodified *P. gingivalis* protein(s) switch TLR2 signaling into the MyD88-independent pathway, thereby modulating host immune responses. This possibility should be addressed experimentally in cellular infection models.

We showed that simultaneous occurrence of both citrullination and fimbriae is essential for host cell responses to *P. gingivalis*. However, comparing our observations with the available literature suggests that the role of the interaction between these two factors is highly strain- and host cell type-dependent. For example, PPAD deficiency in the ATCC 33277 strain has no effect on its ability to incorporate into multispecies biofilms (10), whereas deletion of PPAD from *P. gingivalis* strain 381, which is closely related to ATCC 33277, increases its biofilm-forming potential (42). Similar heterogeneous effects of citrullination on bacterial invasiveness have been observed. While the ability of PPAD-deficient ATCC 33277 strains to adhere to and invade PHGFs is impaired significantly (9), inactivation or deletion of PPAD has no effect on *P. gingivalis* adhesion to and invasion of keratinocytes (10). It should be noted that the hTERT-immortalized gingival keratinocyte (TIGK) cell line was used in that study (10), and we have shown previously that antimicrobial responses of immortalized cell lines can be disrupted by epigenetic defects (25). However, expression of TLRs and susceptibility to *P. gingivalis* invasion are comparable in TIGKs and parental cells (50), suggesting that these results reflect the behavior of primary cells, and that citrullination-dependent differences in *P. gingivalis* invasiveness are indeed cell type-specific.

The reporter cell lines and PHGFs used in our study were largely non-responsive to infection with *P. gingivalis* W83 and clinical isolates. This is surprising since the W83 strain induced a TLR2-dependent immune response in a mouse model of infection (51). This discrepancy could be explained by the presence of a more complex repertoire of receptors and co-receptors on immune cells compared to cell types used in our experiments. The presence of co-receptors and accessory proteins reduces the threshold of the ligand concentrations needed for receptor activation (30). It is therefore possible that the specific experimental conditions used in our assays did not lead to detectable readouts despite the ability of these strains to activate cells. Indeed, *P. gingivalis* W83 and clinical isolates did induce inflammatory cytokine release by PHGFs, albeit to a much lesser extent than the ATCC 33277 strain (data not shown). It should also be noted that, in addition to fimbriae, sphingolipids (52, 53), serine dipeptide lipids (53) and glycine lipids (54) of *P. gingivalis* are TLR2 agonists. Therefore, it is likely that host cells are activated by multiple bacterial ligands and virulence factors in a strain-specific manner.

The immense variability of fimbriae and other *P. gingivalis* virulence factors (55) is the possible reason of strain-dependent activation of TLR2 in a citrullination-dependent manner. First, *P. gingivalis* strains express one of six allelic variants of the *fimA* gene (37, 56). We found that most of the tested clinical strains were weak activators of TLR2. Importantly, most carried type II and IV FimA in line with clinical data showing that these types of FimA are the most prevalent fimbriae genotypes detected in periodontitis patients (57–59). Gene swap studies show that expression of type II (60, 61) and IV (62) fimbriae correlates with increased adhesion and invasiveness of *P. gingivalis*, while type I fimbriae-bearing isolates are more virulent (60). Our data reveal striking differences in FimA production between ATCC 33277 and clinical isolates, in line with a study demonstrating that various *P. gingivalis* strains differ with respect to FimA expression, and that their binding activity depends on both the expression level and type of FimA (63). Second, the fimbriae operon is regulated by the FimS/FimR two component system, and defects in the FimS histidine sensor kinase lead to an absence of fimbriae (33), which is the case in the W83 strain. In our experiments, W83 had a very weak TLR2-activating potential, which is consistent with the lack of fimbriae on its surface. Third, *P. gingivalis* has the ability to exchange *fimA* alleles between various strains *via* natural competence (62). Fourth, the accessory fimbriae subunits can appear in two distinct types, and FimCDE type I is associated with type IV FimA (55). These observations, together with the results of our study, highlight the complexity of mechanisms that enable *P. gingivalis* to adapt to and persist in the host environment.

Collectively, the data presented herein demonstrate the importance of citrullination of *P. gingivalis* proteins in the context of TLR2-dependent host signaling and bacterial virulence. We also demonstrate striking differences between various *P. gingivalis* strains with respect to their potential to activate host proinflammatory responses, especially through TLR2. Collectively, these results are an important step towards uncovering a new mechanism used by *P. gingivalis* to manipulate host responses. While our data provide strong evidence that fimbriae are the key *P. gingivalis* virulence factors regulated by PPAD, the potential citrullination sites on fimbriae components (or other proteins involved in fimbriae assembly) remain to be determined. Potential crosstalk between different host cells receptors activated in a citrullination-dependent manner and specific signaling pathways activated in response to citrullinated fimbriae (or other bacterial proteins) also require further characterization. Finally, future studies will need to delineate the role of this additional layer of virulence regulation from the perspective of bacterial fitness in the host environment. A better understanding of the molecular mechanisms underlying *P. gingivalis* virulence and its sophisticated interaction with the host immune system will be critical for developing new therapeutic strategies for periodontitis and associated comorbidities.

## Data Availability Statement

The original contributions presented in the study are included in the article/**Supplementary Material**. Further inquiries can be directed to the corresponding authors.

## Ethics Statement

The studies involving human participants were reviewed and approved by Bioethical Committee of the Jagiellonian University. The patients/participants provided their written informed consent to participate in this study.

## Author Contributions

Conceived and designed the experiments: AW, AMG, and JP. Performed the experiments: AW, GPB, and KŁ-Ć. Analyzed the data: AW, AMG, and JP. Contributed to reagents/materials/analysis tools: SE and RJL. Wrote the paper: AW, AMG, and JP. Critically revised the manuscript: AW, GPB, RJL, AMG, and JP. All authors read and approved the submitted version of the manuscript.

## Funding

AW is supported by research grant from Faculty of Biochemistry, Biophysics and Biotechnology, Jagiellonian University, Krakow, Poland – Grant for Young Researchers (grant number MNS 3/2020); GPB by grant from National Science Centre of Poland (grant number 2018/29/N/NZ1/00992); AMG by grants from the Foundation for Polish Science (FIRST TEAM program co-financed by the European Union under the European Regional Development Fund; grant number POIR.04.04.00-00-5EDE/18-00) and National Science Centre of Poland (grant number 2019/35/B/NZ5/01823); JP by grants from National Science Centre of Poland (grant number 2018/30/A/NZ5/00650 and US National Institutes of Health (NIDCR, DE 022597); RJL by DE011111 and DE012505 from NIH/NIDCR.

## Conflict of Interest

The authors declare that the research was conducted in the absence of any commercial or financial relationships that could be construed as a potential conflict of interest.

## Publisher’s Note

All claims expressed in this article are solely those of the authors and do not necessarily represent those of their affiliated organizations, or those of the publisher, the editors and the reviewers. Any product that may be evaluated in this article, or claim that may be made by its manufacturer, is not guaranteed or endorsed by the publisher.
