# Corrigendum: TLR2 activation by *Porphyromonas gingivalis* requires both PPAD activity and fimbriae

**DOI:** 10.3389/fimmu.2024.1476001

**Published:** 2024-08-15

**Authors:** Aleksandra Wielento, Grzegorz P. Bereta, Katarzyna B. Łagosz-Ćwik, Sigrun Eick, Richard J. Lamont, Aleksander M. Grabiec, Jan Potempa

**Affiliations:** ^1^ Department of Microbiology, Faculty of Biochemistry, Biophysics and Biotechnology, Jagiellonian University, Krakow, Poland; ^2^ Department of Periodontology, Laboratory of Oral Microbiology, School of Dental Medicine, University of Bern, Bern, Switzerland; ^3^ Department of Oral Immunology and Infectious Diseases, School of Dentistry, University of Louisville, Louisville, KY, United States

**Keywords:** *Porphyromonas gingivalis*, citrullination, fimbriae, TLR2, reporter cell lines, gingival fibroblasts, peptidylarginine deiminase, inflammation

In the published article, there was an error in the Funding statement. An incorrect number (2018/31/B/NZ1/03968) was used to acknowledge the JP’s grant from National Science Centre of Poland.

AW is supported by research grant from Faculty of Biochemistry, Biophysics and Biotechnology, Jagiellonian University, Krakow, Poland – Grant for Young Researchers (grant number MNS 3/2020); GPB by grant from National Science Centre of Poland (grant number 2018/29/N/NZ1/00992); AMG by grants from the Foundation for Polish Science (FIRST TEAM program co-financed by the European Union under the European Regional Development Fund; grant number POIR.04.04.00-00-5EDE/18-00) and National Science Centre of Poland (grant number 2019/35/B/NZ5/01823); JP by grants from National Science Centre of Poland (grant number 2018/31/B/NZ1/03968) and US National Institutes of Health (NIDCR, DE 022597); RJL by DE011111 and DE012505 from NIH/NIDCR.

The correct Funding statement appears below.

AW is supported by research grant from Faculty of Biochemistry, Biophysics and Biotechnology, Jagiellonian University, Krakow, Poland – Grant for Young Researchers (grant number MNS 3/2020); GPB by grant from National Science Centre of Poland (grant number 2018/29/N/NZ1/00992); AMG by grants from the Foundation for Polish Science (FIRST TEAM program co-financed by the European Union under the European Regional Development Fund; grant number POIR.04.04.00-00-5EDE/18-00) and National Science Centre of Poland (grant number 2019/35/B/NZ5/01823); JP by grants from National Science Centre of Poland (grant number 2018/30/A/NZ5/00650 and US National Institutes of Health (NIDCR, DE 022597); RJL by DE011111 and DE012505 from NIH/NIDCR.

The authors apologize for this error and state that this does not change the scientific conclusions of the article in any way. The original article has been updated.

