# Supplementary material for: TLR2 Activation by Porphyromonas gingivalis Requires Both PPAD Activity and Fimbriae
Source: Front Immunol. 2022 Apr 1;13:823685. doi: 10.3389/fimmu.2022.823685 (PMC9010743; doi:10.3389/fimmu.2022.823685)
Supplement: Supplementary file 1 [file DataSheet_1.pdf]

\Supplementary Material

1.

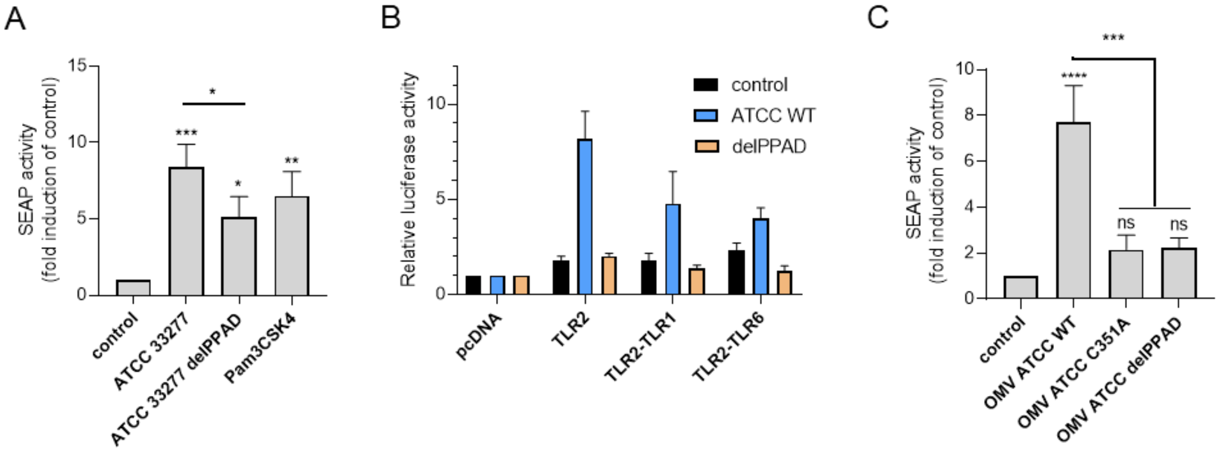

Supplementary figure 1. PPAD expression and activity is required for TLR2 as well as TLR2 heterodimers (TLR2-TLR1 and TLR2-TLR6) activation. HEK Blue cells overexpressing TLR2 receptor were (A) infected for 4 h (MOI=100) with the ATCC 33277 strain and its isogenic PPAD mutant (delPPAD), n=3 or (C) stimulated with outer membrane vesicles (OMV) isolated from the ATCC 33277 strain or its isogenic PPAD mutants, n=3. Results are presented as fold induction relative to control (unstimulated) cells. Results were compared with those from ATCC 33277 strain. Mean ± SD, * $p<0.05$; *** $p<0.001$; **** $p<0.0001$; ns- no statistical significance. 1-way ANOVA followed by Tukey's multiple comparisons test. (B) U251 MG cells overexpressing various dimers of TLR2 (homodimer -TLR2 and heterodimers: TLR2-TLR1, TLR2-TLR6) were infected for 4 h with the ATCC 33277 (ATCC WT) strain or the isogenic PPAD mutant strain (MOI=100). Results are presented as a ratio of *Firefly* luciferase activity to β-galactosidase activity and are normalized to cells infected with the same strain and transfected with an empty vector (pcDNA), n=3. Mean ± SEM.

2.

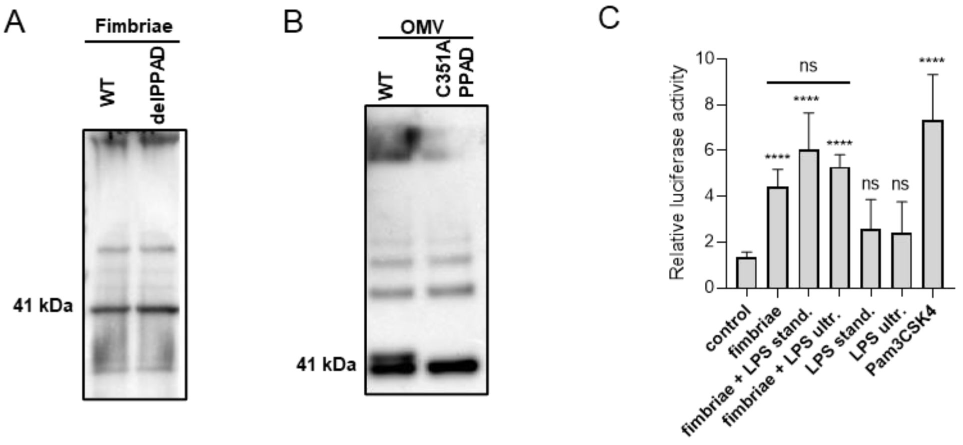

Supplementary figure 2. Fimbriae and OMV from WT-*P. gingivalis* ATCC 33277 (WT) and PPAD mutant strains contain comparable amounts of FimA. Western blot analysis of FimA (41 kDa monomer) protein levels in (A) fimbriae isolated from WT ATCC 33277 (WT) and the PPAD mutant (delPPAD) and (B) outer membrane vesicles isolated from WT ATCC 33277 (WT) and the catalytic PPAD mutant (C351A PPAD) ATCC 33277 strains. **(C) TLR2 is activated by the purified ATCC 33277 FimA protein, but addition of *P. gingivalis* LPS has no effect on TLR2 activation.** U251 MG cells overexpressing TLR2 were treated for 4 h with standard (stand.)/ultrapure (ultr.) LPS preparations alone or in combination with wild-type fimbriae (35 µg/ml), n=3-6. Pam3CSK4 was used as a positive control. Results are presented as the ratio of *Firefly* luciferase activity to β-galactosidase activity and are normalized to those from cells stimulated/infected with the same factor and transfected with an empty vector. Mean + SD; **** $p<0.0001$; ns, no statistical significance. 1-way ANOVA followed by Tukey's multiple comparisons test.
